# Predictive value of preoperative CT enhancement rate and CT perfusion parameters in colorectal cancer

**DOI:** 10.1186/s12876-024-03257-0

**Published:** 2024-05-21

**Authors:** Ze-mao Li, Wei Zhou, Li Feng, Hui-ying Zhang, Wei-bin Chen

**Affiliations:** 1 North China University of Science and Technology Affiliated Hospital, Tangshan, Hebei 063000 China; 2 North China University of Science and Technology, Tangshan, Hebei 063000 China

**Keywords:** CT perfusion imaging, Colorectal cancer, Tumor angiogenesis, Tumor markers

## Abstract

**Background:**

Angiogenesis is a critical step in colorectal cancer growth, progression and metastasization. CT are routine imaging examinations for preoperative clinical evaluation in colorectal cancer patients. This study aimed to investigate the predictive value of preoperative CT enhancement rate (CER) and CT perfusion parameters on angiogenesis in colorectal cancer, as well as the association of preoperative CER and CT perfusion parameters with serum markers.

**Methods:**

This retrospective analysis included 42 patients with colorectal adenocarcinoma. Median of microvessel density (MVD) as the cut-off value, it divided 42 patients into high-density group (MVD ≥ 35/field, *n* = 24) and low-density group (MVD < 35/field, *n* = 18), and 25 patients with benign colorectal lesions were collected as the control group. Statistical analysis of CER, CT perfusion parameters, serum markers were performed in all groups. Receiver operating curves (ROC) were plotted to evaluate the diagnostic efficacy of relevant CT perfusion parameters for tumor angiogenesis; Pearson correlation analysis explored potential association between CER, CT perfusion parameters and serum markers.

**Results:**

CER, blood volume (BV), blood flow (BF), permeability surface (PS) and carbohydrate antigen 19 − 9 (CA19-9), carbohydrate antigen 125 (CA125), carcinoembryonic antigen (CEA), trefoil factor 3 (TFF3), vascular endothelial growth factor (VEGF) in colorectal adenocarcinoma were significantly higher than those in the control group, the parameters in high-density group were significantly higher than those in the low-density group (*P* < 0.05); however, the time to peak (TTP) of patients in colorectal adenocarcinoma were significantly lower than those in the control group, and the high-density group showed a significantly lower level compared to the low-density group (*P* < 0.05). The combined parameters BF + TTP + PS and BV + BF + TTP + PS demonstrated the highest area under the curve (AUC), both at 0.991. Pearson correlation analysis showed that the serum levels of CA19-9, CA125, CEA, TFF3, and VEGF in patients showed positive correlations with CER, BV, BF, and PS (*P* < 0.05), while these indicators exhibited negative correlations with TTP (*P* < 0.05).

**Conclusions:**

Some single and joint preoperative CT perfusion parameters can accurately predict tumor angiogenesis in colorectal adenocarcinoma. Preoperative CER and CT perfusion parameters have certain association with serum markers.

## Background

The incidence and mortality of colorectal cancer are increasing year by year, many colorectal cancer patients are already at an advanced stage at diagnosis, and have had distant tissue or organ metastasis by the time they are diagnosed. Therefore, it is essential to pre-operatively diagnose and evaluate patients with colorectal cancer. Some previous studies have confirmed that malignant tumor growth, progression, and metastasis depend on adequate blood supply [1]. As a result, the structure and function of tumor neovascularization are imperfect, which tumor cells can effectively penetrate tumor blood vessel endothelial cells. Therefore, angiogenesis is fundamental for the progression, invasion, and metastasis of tumors. Microvessel density(MVD) has become the morphological gold standard to assess angiogenesis in human tumors [2]. MVD can not only evaluate the occurrence and development of various types of malignant tumors, but also is an important indicator for forecasting prognosis [3]. MVD count is a means of assessing the blood supply of solid tumors by calculating the number of angiogenesis shown on various IHC staining [4]. In general, MVD assessment can only be performed after tumor resection, so it is difficult to assess tumor angiogenesis before operation. With the development of science and technology and image post-processing equipment, CT perfusion imaging can obtain the time-density curve (TDC) through continuous CT scanning of the region of interest (ROI) during rapid intravenous injection of contrast agent, and calculate various perfusion parameters to quantitatively evaluate the blood vessels of tumor tissues and indirectly reflect tumor angiogenesis in a non-invasive manner [5]. Serum vascular endothelial growth factor (VEGF) and trefoil factor 3 (TFF3) can promote the growth of blood vessels, which are conducive to the tumor proliferation and spreading. Studies have shown that the VEGF expression of a malignant tumor is higher than that of a benign tumor [6]. At present, the correlation between CT perfusion imaging parameters and MVD has been explored by many researchers at home and abroad, but the predictive value of CT perfusion parameters for MVD is still unclear, and the association of CT perfusion parameters with tumor markers and neovascular markers is still unclear. Based on the above reasons, the purpose of this study is to explore the clinical evaluation value of preoperative CER and CT perfusion parameters for colorectal cancer angiogenesis, as well as the association of CER and CT perfusion parameters with serum tumor markers and tumor neovascular markers.

## Methods

### Patient population

Forty-two patients who were pathologically diagnosed with colorectal adenocarcinoma by preoperative colonoscopy or surgery in our hospital from May 2019 to February 2021 were collected. These cases were grouped according to the MVD determined by IHC. The median MVD (35/field) was used as the cutoff point for grouping, patients were divided into a high density group (MVD ≥ 35/field, *n* = 24) and a low density group (MVD < 35/field, *n* = 18). At the same time, 25 patients with pathologically confirmed benign colorectal lesions were collected as the control group. The inclusion criteria were as follows: (1) Patients had chronic diarrhea, chronic constipation, bloody mucopurulent stool, and other symptoms; (2) The patient was pathologically diagnosed with colorectal cancer who underwent preoperative colonoscopic forceps and postoperative pathological findings; (3) The patient had not received any chemoradiotherapy or other relevant treatment before operation; (4) All patients signed the informed consent before examination. The exclusion criteria were as follows: (1) The patient was allergic to iodine contrast (*n* = 0); (2) The patient had severe liver and renal impairment and severe cardiovascular and cerebrovascular disease (*n* = 0); (3) The image quality fails to meet perfusion (*n* = 0). In the end, there were 18 males and 6 females in the high density group, 14 males and 4 females in the low density group, 17 males and 8 females in the control group. The general clinical data (such as gender, age, etc.) did not vary significantly among the groups (*P* > 0.05 each), which were comparable. See Tables 1 and 2.

**Table 1:** Comparison of general clinical data

Indicators		High density group	Low density group	Control group	χ^2^/F	*P*
Male /Female		18/6	14/4	17/8	0.187	0.911
Age (years)		63.92 ± 5.20	62.83 ± 5.62	66.64 ± 5.56	2.931	0.061
Height (cm)		174.0 ± 6.85	173.5 ± 6.53	171.6 ± 7.09	0.822	0.444
Weight (kg)		63.29 ± 6.46	63.94 ± 6.58	63.61 ± 6.53	0.052	0.949
pathologic staging	T_1 − 2_	7	5	-	0.010	0.922
	T_3 − 4_	17	13			

**Table 2:** Distribution of sites in 42 patients with colorectal adenocarcinoma

Groups	Right colon	Left colon	Sigmoid colon	Rectosigmoid junction	Rectum	χ^2^	*P*
High density group	4	2	6	4	8	0.453	0.978
Low density group	2	1	5	3	7		

### Scanning methods and parameters of CT

All patients fasted for 6 h before the CT scanning. Examination equipment: GE Revolution CT. All metal foreign bodies that may affect the scanning inspection shall be excluded before the inspection. The patients were lying flat on the examination bed. The CT scanning scheme includes plain CT scan and dynamic contrast-enhanced CT scan. Plain CT scan was obtained to confirm the scanning range of perfusion, CT perfusion scan was performed with a scan range from 3.5 cm above the upper border of lesions to 3.5 cm below the lower border of lesions. After plain scanning, 65 ml iohexol was injected intravenously through the right elbow at a flow rate of 3.5 ml/s using German ORICH high-pressure syringe. Dynamic scans were performed 10s after injection of iodine contrast started, subjects were scanned every 10s for 25 phases. The scanning parameters were as follows: tube voltage: 120KV; tube current: 200mAs; scanning layer thickness: 5.0 mm; reconstruction thickness: 5.0 mm; pitch: 1.0; matrix: 512 × 512.

### CT perfusion image processing

**Fig. 1:**
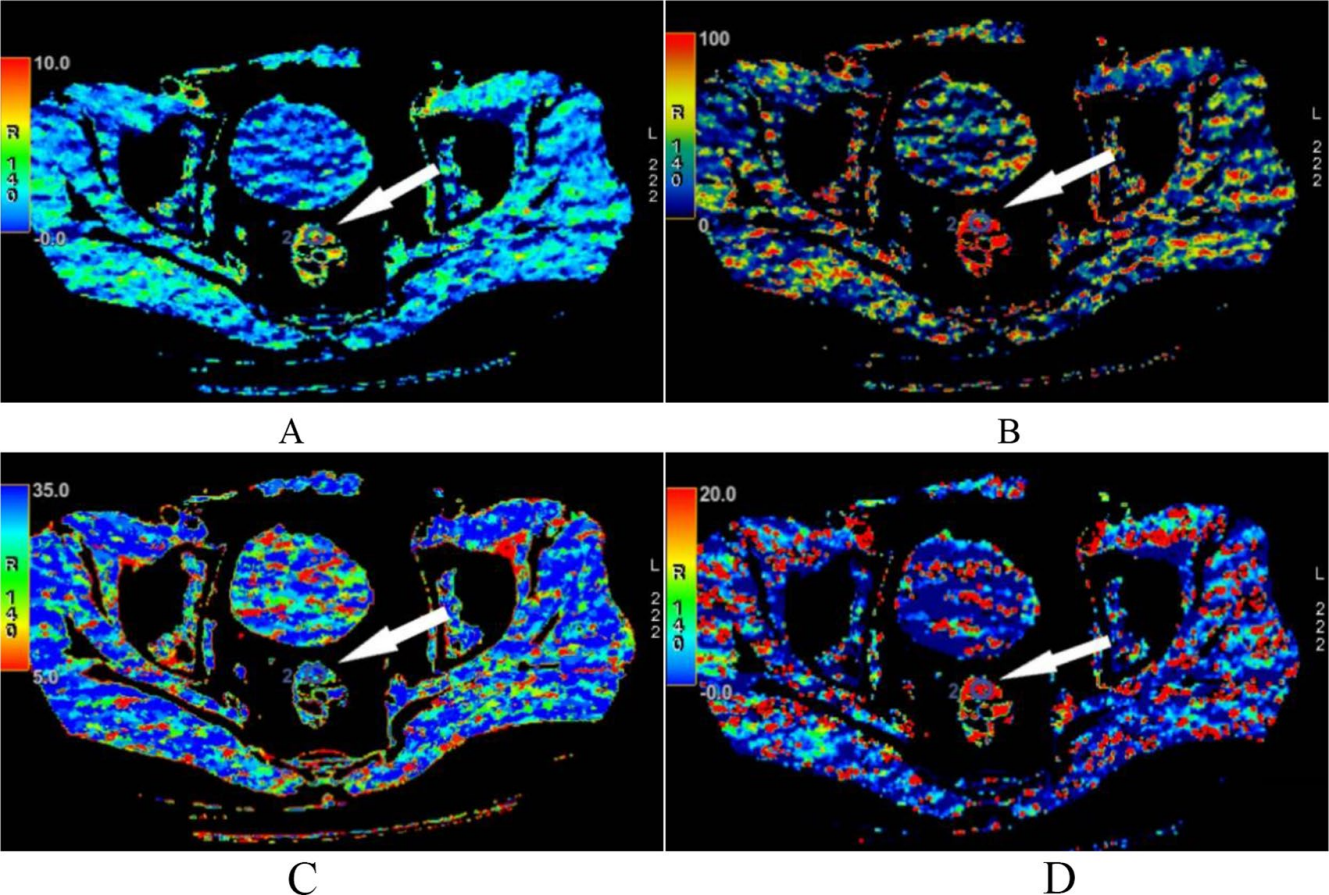
BV (**A**), BF (**B**), TTP (**C**) and PS (**D**) were randomly selected from patients with colorectal cancer. **A**: BV diagrams of patients with colorectal cancer were randomly selected (shown by an arrow), BV of the selected ROI was increased; **B**: BF diagrams of patients with colorectal cancer were randomly selected (shown by an arrow), BF of the selected ROI was significantly increased; **C**: TTP diagrams of patients with colorectal cancer were randomly selected (shown by an arrow), TTP of the selected ROI was significantly decreased; **D**: PS diagrams of patients with colorectal cancer were randomly selected (shown by an arrow), PS of the selected ROI was significantly increased

All images were transferred to a workstation (AW4.7) for processing. Enter the module of CT perfusion 4D, select the mode of CT body tumor perfusion, use deconvolution method, select the artery on the largest level of the lesion to generate Review Permeability Maps, and measure parameters in color map of the Review Permeability MVD Maps. Referring to pathology, place ROI in the lesion area, select the maximum cross-sectional area of the lesion for measurement through MPR reconstruction, select the solid part, and avoid selecting blood vessels, necrotic liquefaction areas, gases, and fats; The ROI placement of benign lesions is based on pathological results, and the maximum cross-section of the lesion axis or long axis is selected for measurement through MPR reconstruction. Special attention should be paid to avoiding interference factors such as gas, fat, and intestinal contents. When the intestinal wall is thin and difficult to measure, the image can be enlarged for measurement. BV, BF, TTP, Mean transit time (MTT), PS were obtained. The maximum level of tumors were outlined into ROI in plain scan and arterial phase to obtain the CT value, and calculate the arterial phase enhancement ratio. Enhancement ratio = (maximum CT value of enhanced scan - the value of plain scan)/the CT value of plain scan×100%. The ROI drawing and parameters were tested in triplicate and the average values were used for final data analysis. See Fig. 1.

### Measurement of serum tumor biological indicators and neovascular markers

 Determination of tumor serum biological indicators: 2 ml fasting peripheral venous blood was collected from each group of patients, placed at room temperature for 30 min, and clarified by low speed centrifugation at 4℃. The content of tumor markers, including carbohydrate antigen 19 − 9 (CA19-9), carbohydrate antigen 125 (CA125) and carcinoembryonic antigen (CEA), were determined by enzyme-linked immunosorbent assay.

Determination of serum tumor markers of tumor neoangiogenesis: Peripheral venous blood samples of patients in each group were routinely collected in the morning, the samples were fully coagulated at low temperature, after centrifugation at low temperature, the supernatants were collected. The serum levels of TFF3 and VEGF were measured by enzyme-linked immunosorbent assay.

### Measurement of microvessel density (MVD)

**Fig. 2:**
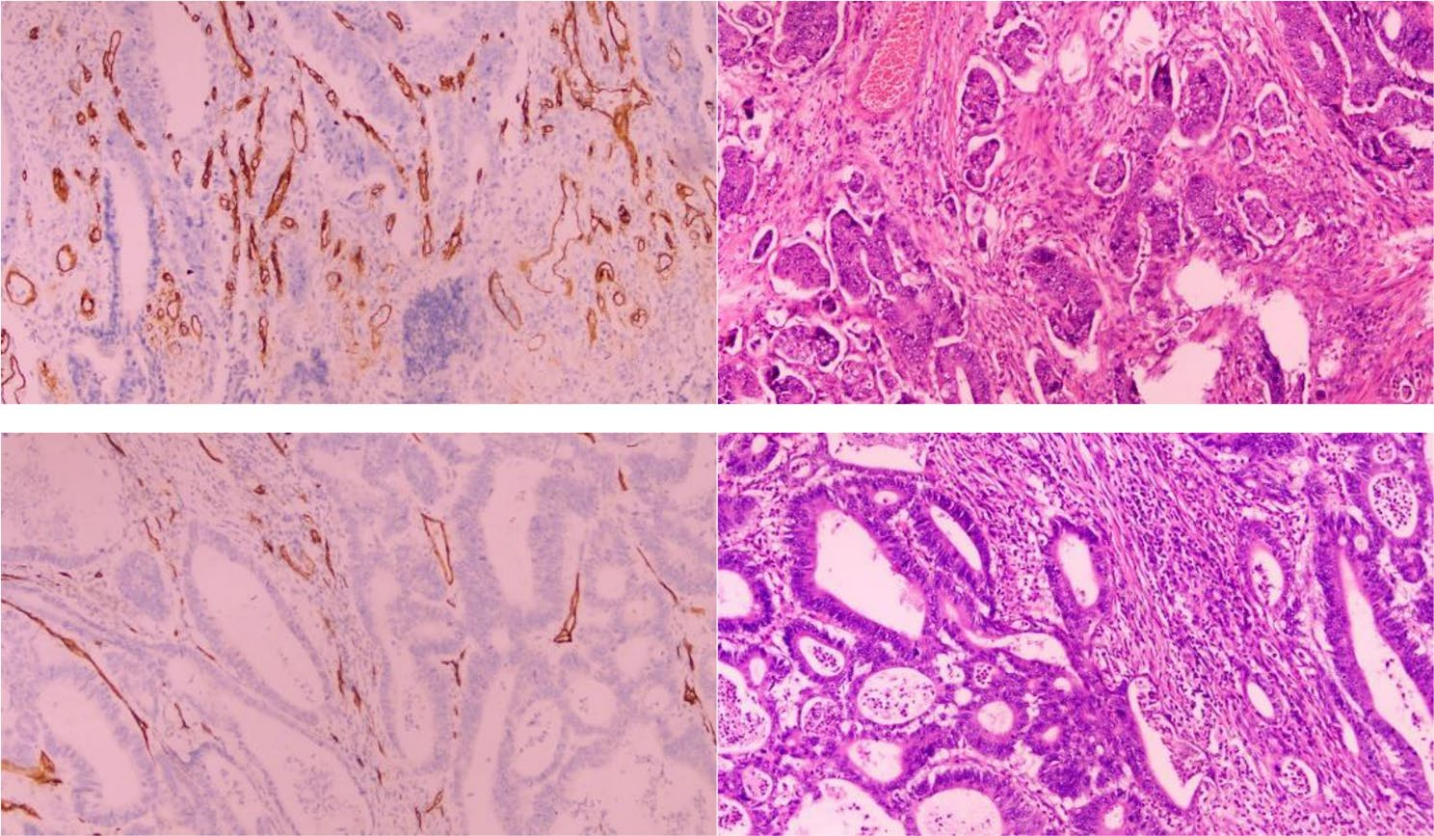
CD34 positive microvascular proliferation can be observed among cancer cell nests. (The above two images show high MVD, on the right is HE staining; The under two images show low MVD, on the right is HE staining)

The postoperative tissue samples were determined by immunohistochemical staining of CD34. Weidner counting method was used, the whole tissue sections were scanned under low magnification to find the visual fields with clear staining of endothelial cells and tumor cells, well-background control, the densest number of microvessels, and the largest distribution of tumor cells [7]. Five distinct visual fields (×200) were randomly chosen. The microvessels were counted, and the mean count of microvessels of all patients was 35.01 ± 1.64 per mm^2^. The mean value (35/mm^2^) was taken as the cut-off value. Those ≥ 35/mm^2^ were divided into high-density group and those < 35/mm^2^ were divided into low-density group. See Fig. 2.

### Statistical methods

SPSS 25.0 statistical software was used for data analysis. The comparison of count data was performed using Chi-square tests. Measurement data were compared with one-way analysis of variance, and the comparison between groups was performed by LSD-t test. The parameters with a p-value less than 0.05 were included in logistic regression analysis. Pearson correlation was utilized for correlation analysis. *P* < 0.05 was considered statistically significant. GraphPad Prism 8 software was used to draw forest plots.

## Results

### Comparison of CER and CT perfusion parameters among three groups

Patients with colorectal adenocarcinoma had higher CER, BV, BF, PS levels than controls, the high density group was more significantly increased than the low density group (*P* < 0.05). TTP of patients with colorectal cancer was lower than controls, the high density group was more significantly decrease than the low density group (*P* < 0.05). There were no significant differences in MTT among the three groups (*P* > 0.05). See Table 3.

**Table 3:** Comparison of CER and CT perfusion parameters among the three groups

Groups (*n*)	BV(mL/100 g)	BF[ml/(100 g·min)]	TTP(s)	MTT(s)	PS[ml/(100 g·min)]	CER
High density group (24)	7. 65 ± 1.36	67. 33 ± 12.16	20. 78 ± 4.18	11. 54 ± 2.22	34.25 ± 6. 65	0.87 ± 0.03
Low density group (18)	6. 73 ± 1.29	52. 84 ± 11.43	28. 70 ± 4.99	11. 64 ± 2.09	23.33 ± 3.02	0.71 ± 0.02
Control group (25)	4. 80 ± 1.28	38. 42 ± 10.42	42. 61 ± 5.38	12. 02 ± 2.51	12. 21 ± 4.63	0.51 ± 0.03
F	30.242	39.850	125.912	0.297	113.046	1075.508
*P*	<0.001	<0.001	<0.001	0.744	<0.001	<0.001

**Fig. 3:**
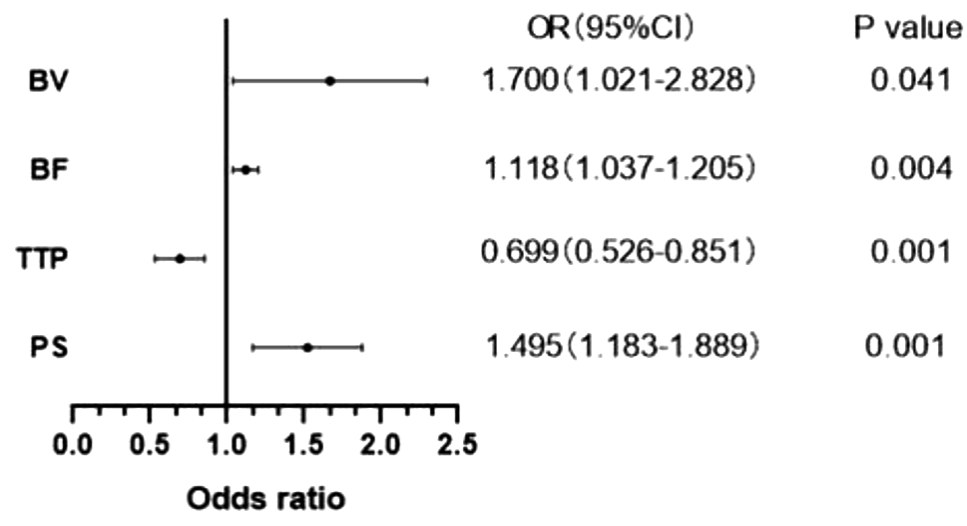
Forest plot for logistic regression analysis of CER and CT perfusion parameters in high and low density groups

Logistic regression was used to analyze the above statistically significant CT parameters(CER, BV, BF, TTP, PS), and CT perfusion parameters(BV, BF, TTP, PS) turned out to have statistically significant difference (*P* < 0.05). Higher the MVD counts indicated the higher probability of the higher BV, BF and PS values; higher the MVD counts indicated the higher probability of the lower TTP values. See Table 4; Fig. 3.

**Table 4:** Logistic regression analysis of CER and CT perfusion parameters in high and low density groups

Parameters	*P*	OR	95%CI
CER	0.996	3.961	1.526∼5.071
BV	0.041*	1.700	1.021∼2.828
BF	0.004*	1.118	1.037∼1.205
TTP	<0.001*	0.699	0.526∼0.851
PS	<0.001*	1.495	1.183∼1.889

**Fig. 4:**
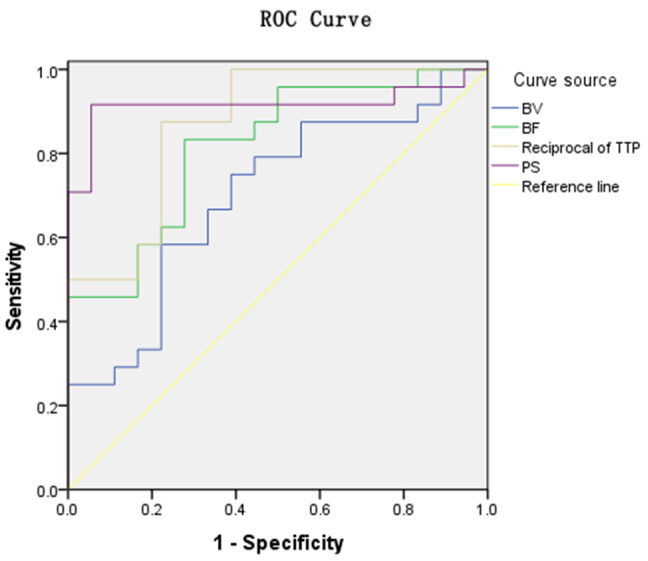
ROC curves of CT perfusion parameters for high and low MVD counts in colorectal cancer

**Fig. 5:**
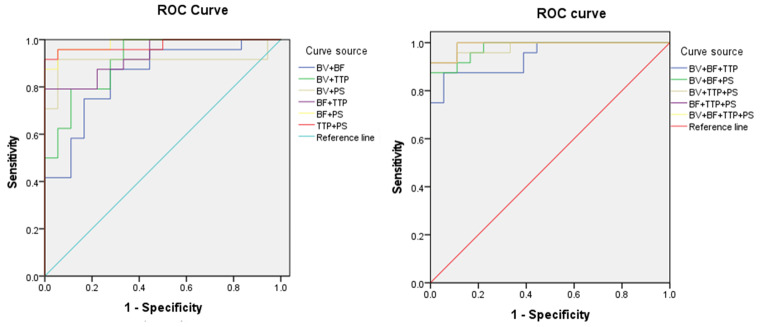
ROC curves of CT perfusion parameters for high and low MVD counts in colorectal cancer

Logistic regression was used to obtain statistically significant parameters and joint parameters, which were then used for ROC curves. The ROC curves were plotted, and AUC values for each parameter and joint parameters were calculated and compared. The ROC curves showed that TTP and PS had the higher AUC, at 0.873 and 0.917, respectively; the diagnostic thresholds of TTP and PS were 24.29s, 27.83 ml/(min·100 g), respectively; the sensitivity were 81.5%, 91.7%, respectively; the specificity were 77.8% and 94.4%, respectively; and the Youden index were 0.653, 0.861, respectively. See Table 5; Fig. 4. The ROC curves showed that BF + PS, TTP + PS, BF + TTP + PS and BV + BF + TTP + PS had the higher AUC, at 0.984, 0.977, 0.991 and 0.991, respectively, at the same time, the sensitivity, the specificity and the Youden index demonstrated a high level. See Table 6; Fig. 5.

**Table 5:** CT perfusion parameters analyzed the area under the curve with high and low microvascular density counts in colorectal cancer

Parameters	AUC(95%CI)	Threshold(HU)	Sensitivity(%)	Specificity(%)	Youden index
BV	0.699(0.538∼0.831)	7.140	75.0	61.1	0.361
BF	0.817(0.668∼0.919)	55.560	83.3	72.2	0.556
1/TTP	0.873(0.733∼0.955)	0.039	87.5	77.8	0.653
PS	0.917(0.789∼0.979)	27.830	91.7	94.4	0.861

**Table 6:** CT perfusion joint parameters for area analysis of areas under the curve with high and low microvascular density counts in colorectal cancer

Parameters	AUC(95%CI)	Sensitivity(%)	Specificity(%)	Youden index
BV + BF	0.847(0.703∼0.939)	87.5	72.2	0.597
BV + TTP	0.912(0.783∼0.977)	79.2	88.9	0.681
BV + PS	0.910(0.780∼0.976)	91.7	94.4	0.861
BF + TTP	0.931(0.808∼0.986)	79.2	100.0	0.792
BF + PS	0.984(0.887∼1.000)	95.8	94.4	0.902
TTP + PS	0.977(0.875∼0.999)	91.7	100.0	0.917
BV + BF + TTP	0.942(0.824∼0.991)	87.5	94.4	0.819
BV + BF + PS	0.979(0.879∼1.000)	87.5	100.0	0.875
BV + TTP + PS	0.981(0.883∼1.000)	91.7	100.0	0.917
BF + TTP + PS	0.991(0.899∼1.000)	91.7	100.0	0.917
BV + BF + TTP + PS	0.991(0.899∼1.000)	91.7	100.0	0.917

### Comparison of serum tumor neovascularization markers among the three groups

Patients with colorectal adenocarcinoma had significantly higher levels of TFF3, VEGF than controls (*P* < 0.05), the high-density group was more significantly higher than the low-density group (*P* < 0.05), See Table 7.

**Table 7:** Comparison of serum tumor neovascularization markers among the three groups

Groups(*n*)	TFF3(ng / ml)	VEGF(pg / ml)
High density group(24)	47. 35 ± 9.32	467. 84 ± 82.86
Low density group(18)	36. 64 ± 8.53	382. 42 ± 91.69
Control group(25)	24. 23 ± 6.78	201. 22 ± 86.32
F	48.460	60.322
*P*	<0.001	<0.001

### Comparison of serum tumor markers among the three groups

Patients with colorectal adenocarcinoma had significantly higher serum CA19-9, CA125, CEA levels than controls(*P* < 0.05), the high density group was more significantly increased than the low density group (*P* < 0.05), See Table 8.

**Table 8:** Comparison of serum tumor markers among the three groups

Groups(*n*)	CA19-9(g/ml)	CA125(g/ml)	CEA(g/ml)
High density group(24)	45.38 ± 5.41	27.56 ± 3.73	17.87 ± 3.12
Low density group(18)	23.43 ± 3.59	12.63 ± 2.59	8.51 ± 2.87
Control group(25)	8.36 ± 1.25	8.16 ± 1.83	1.94 ± 0.43
F	582.103	308.700	272.422
*P*	<0.001	<0.001	<0.001

### Correlation analysis between CER, CT perfusion imaging parameters and serum tumor neovascular markers and tumor markers

Pearson correlation showed that the CA19-9, CA125, CEA and TFF3 positively correlated with CER, BV, BF and PS (*P* < 0.05), while the CA19-9, CA125, CEA and TFF3 negatively correlated with TTP (*P* < 0.05). See Table 9.

**Table 9:** Correlation Analysis between CER, CT perfusion imaging parameters and serum tumor neovascular markers and tumor markers

parameters	Statistical values	BV	BF	TTP	MTT	PS	CER
CA19-9	r	0.319	0.481	− 0.596	-0.021	0.640	0.863
	*P*	0.039	<0.001	<0.001	0.897	<0.001	<0.001
CA125	r	0.322	0.482	-0.672	0.064	0.671	0.882
	*P*	0.038	<0.001	<0.001	0.685	<0.001	<0.001
CEA	r	0.334	0.566	-0.515	0.011	0.609	0.778
	*P*	0.031	<0.001	<0.001	0.946	<0.001	<0.001
TFF3	r	0.315	0.324	-0.483	0.156	0.387	0.446
	*P*	0.042	0.037	<0.001	0.323	0.011	0.003
VEGF	r	0.300	0.354	-0.399	-0.239	0.396	0.351
	*P*	0.039	0.021	0.035	0.127	0.010	0.023

## Discussion

The intratumoral microvessel density has been regarded as one important indicator for quantitatively analyzing tumor angiogenesis, which can clearly reflect the intratumoral blood vessels state and tumor-induced angiogenesis ability. It is well documented that tumor neovascularization is strongly related to tumor growth and metastasis [8]. Probably its because of the ample vascular capillary network that provide nutrients and oxygen required for tumor tissue invades and migrates to adjacent tissues. CT perfusion imaging can reveal the hemodynamic changes of tumor tissue, and can quantitatively reflect tumor MVD by each perfusion parameter, and it has been used as a tool to estimate the prognosis of patients with tumor following chemotherapy [9]. Therefore, CT perfusion parameters may be suitable proxy indexes for angiogenesis. In this paper, the research results of CT parameters used to evaluate angiogenesis in colorectal cancer show that patients with colorectal cancer had higher CER, BV, BF, PS levels than patients with benign colorectal lesions, the high density group was more significantly increased than the low density group; patients with colorectal cancer had lower TTP levels than controls, the high density group was more significantly decreased than the low density group, so it is easy to conclude that MVD is a significant factor that affects CER and CT perfusion parameters in colorectal cancer. Goh V [10] et al. reported that the tumor BV and PS correlate positively with MVD, which can accurately reflect the MVD in colorectal cancer. Xu Y [11] et al. showed that the higher the BF and the lower the TTP indicating abundant neovascularization in the tumor region, the more the amount of blood flowing through the vessels, the faster the flow velocity and the higher the malignancy of the tumor. Based on the analysis of perfusion parameters of high-density and low-density groups, the following conclusions can be drawn: higher MVD was associated with higher BV, BF, PS; higher MVD was associated with lower TTP, this is consistent with previous research results. BF, TTP, PS, BF + PS, TTP + PS, BF + TTP + PS and BV + BF + TTP + PS have been shown to be useful to estimate MVD by comparison of AUC. The higher value of MVD, the more tumor angiogenesis and a rich blood supply may be considered when BF values exceeding 55.560 ml/min, TTP values below 24.29s, PS values exceeding 27.830 ml/(100 g·min). Therefore, it can be concluded that non-invasive CT perfusion parameters and its joint parameters were used to quantitatively assess the MVD of cancer tissue in colorectal cancer.

Despite the large diversity of vascular growth factor, considering the special role of VEGF in mitosis of tumor, so VEGF plays a crucial role in tumor angiogenesis [12]. TFF3, a member of the trefoil factor family, is a secreted protein. Overexpression of TFF3 is found in many intestinal tumors. TFF3 is able to directly modulate the multiplication cycle on tumor cells, which can promote tumor growth, invasion, angiogenesis and even metastasis [13]. Thielemann A, MacConmara M et al. [14, 15] in their study revealed that a significant correlation was found between the expression of VEGF and MVD in breast cancer patients. In this study, TFF3 and VEGF compared with MVD and the controls are shown that the differences among the factors were statistically significant, which indicates patients with colorectal cancer had higher serum TFF3, VEGF levels than controls, the high-density group had significantly higher TFF3 and VEGF levels than the low-density group. The reason for this may be that blood vessels within the colorectal tumor tissue are sensitive to TFF3 and VEGF, TFF3 and VEGF induce vascular endothelial growth and promote angiogenesis. Tumor vascular permeability can be increased to some extent in the extracellular matrix were degraded under the action of TFF3 and VEGF. TFF3 and VEGF can promote tumor cells to enter the circulation system by the high vascular permeability, and then ended with their seeding and growing in a remote site [16–18]. The results of the present study concerning the association between TFF3, VEGF, and CT parameters demonstrate BV, BF and PS correlate positively with serum TFF3 and VEGF, but TTP correlate inversely with serum TFF3 and VEGF, it is suggested that the levels of serum TTF3 and VEGF are related to the microvascular function in colorectal cancer, and the parameters quantitative analysis were conducted using DSCT perfusion imaging have relevant trends, it suggests that TFF3 and VEGF have potential for clinical non-invasive evaluation of tumor angiogenesis in colorectal cancer.

CA19-9, CA125 and CEA are broad-spectrum tumor markers. Its abnormal expression may be involved in various malignant tumors [19]. CEA rarely presents in normal adults, increased serum level of CEA indicates the presence of malignancy, moreover, a close correlation was detected between the degree of the CEA elevated and rapid proliferative capacity of tumor tissue. CA19-9 and CA125 are tumor markers of carbohydrate antigen, the increase in tumor markers can indicate the occurrence of gastrointestinal malignancies [20]. CEA, CA19-9 and CA125 can make the cancer cells fall off in primary lesions, the free cancer cells can invade adjacent or distant tissues, forming a metastasis or invasion of adjacent tissue. This study detected the above tumor markers and found that patients with colorectal cancer had higher CEA, CA19-9, CA125 levels than patients with benign colorectal lesions, and CEA, CA19-9, CA125 increase were more evident in high-density group. Pearson correlation analysis showed a positive correlation between serum CA19-9, CA125, CEA and CER, BV, BF, PS (*P* < 0.05), which suggested a correlation between the tumor markers and the CT perfusion parameters, however, the number of cases included in the current study is relatively small, and there is a need to further expand the sample size in future research.

The limitation of this study including the following aspects: The number of cases included in the current study is relatively small, mainly considering the effects of radiation dose and contrast agent dosage during CT perfusion on renal function in patients, this study only analyzed patients with fixed body weight, which may lead to a certain deviation in the results of this study. Future studies also need to expand the sample size, and more meticulous studies need to be performed to accurately define the association between the tumor markers and the CT perfusion parameters. During the lesion measurement process, only two-dimensional measurements of the largest tumor layer were taken, and the representativeness of ROI is limited to a certain extent. In addition, due to the influence of intestinal wall thickness, although strict quality control was implemented in this study, the accuracy of ROI delineation for normal intestinal walls still has certain limitations. Given the limitations mentioned above, future research is needed to explore more scientific and reproducible ROI delineation methods, further comparative studies of different imaging methods, and radiomics studies.

## Conclusion

To conclude, preoperative CT perfusion parameters and combined parameters can accurately predict tumor angiogenesis in colorectal cancer. CER and CT perfusion parameters have a certain correlation with serum tumor markers and tumor neovascularization markers, which can provide a certain reference for clinical prediction of tumor biological behavior.

## Data Availability

The datasets used and/or analyzed during the current study are available from the corresponding author on reasonable request.

## Abbreviations

MVD: Microvessel density
CER: Contrast enhancement ratio
BV: Blood volume
BF: Blood flow
TTP: Time to Peak
MTT: Mean Transit Time
PS: Surface Permeability
CA19-9: Carbohydrate antigen 19 − 9
CA125: Carbohydrate antigen 125
CEA: Carcinoembryonic antigen
TFF3: Trefoil factor 3
VEGF: Vascular endothelial growth factor
OR: Odds ratio

## References

[1] Sun H, Xu Y, Yang Q, Wang W (2014). Assessment of tumor grade and angiogenesis in colorectal cancer: whole-volume perfusion CT. Acad Radiol.

[2] Hawighorst H, Knapstein PG, Knopp MV, Weikel W, Brix G, Zuna I (1998). Uterine cervical carcinoma: comparison of standard and pharmacokinetic analysis of time-intensity curves for assessment of tumor angiogenesis and patient survival. Cancer Res.

[3] Miyata Y, Sakai H (2015). Reconsideration of the clinical and histopathological significance of angiogenesis in prostate cancer: usefulness and limitations of microvessel density measurement. Int J Urol.

[4] Goddard JC, Sutton CD, Furness PN, Kockelbergh RC, O’Byrne KJ (2002). A computer image analysis system for microvessel density measurement in solid tumours. Angiogenesis.

[5] Feng ST, Sun CH, Li ZP, Mak HK, Peng ZP, Guo HY (2010). Evaluation of angiogenesis in colorectal carcinoma with multidetector-row CT multislice perfusion imaging. Eur J Radiol.

[6] Wang J, Tang Z, Wang S, Zeng W, Qian W, Wu L (2016). Differential diagnostic value of computed tomography perfusion combined with vascular endothelial growth factor expression in head and neck lesions. Oncol Lett.

[7] Weidner N, Folkman J, Pozza F, Bevilacqua P, Allred EN, Moore DH (1992). Tumor angiogenesis: a new significant and independent prognostic indicator in early-stage breast carcinoma. J Natl Cancer Inst.

[8] Lazăr D, Tăban S, Raica M, Sporea I, Cornianu M, Goldiş A (2008). Immunohistochemical evaluation of the tumor neoangiogenesis as a prognostic factor for gastric cancers. Rom J Morphol Embryol.

[9] Zhang CQ, Yang S, Zhang LJ, Ma JN, Chen Q (2022). A cohort study to evaluate the efficacy and Value of CT Perfusion Imaging in patients with metastatic osteosarcoma after Chemotherapy. Comput Math Methods Med.

[10] Goh V, Halligan S, Daley F, Wellsted DM, Guenther T, Bartram CI (2008). Colorectal tumor vascularity: quantitative assessment with multidetector CT–do tumor perfusion measurements reflect angiogenesis?. Radiology.

[11] Xu Y, Sun H, Song A, Yang Q, Lu X, Wang W (2015). Predictive significance of Tumor Grade using 256-Slice CT whole-tumor perfusion imaging in colorectal adenocarcinoma. Acad Radiol.

[12] Ash L, Teknos TN, Gandhi D, Patel S, Mukherji SK (2009). Head and neck squamous cell carcinoma: CT perfusion can help noninvasively predict intratumoral microvessel density. Radiology.

[13] Shuford RA, Cairns AL, Moaven O (2020). Precision approaches in the management of Colorectal Cancer: current evidence and latest advancements towards individualizing the treatment. Cancers (Basel).

[14] Thielemann A, Kopczyński Z, Filas V, Breborowicz J, Grodecka-Gazdecka S, Baszczuk A (2008). The determination of VEGF and MVD, among patients with primary breast cancer. Pathol Oncol Res.

[15] MacConmara M, O’Hanlon DM, Kiely MJ, Connolly Y, Jeffers M, Keane FB (2002). An evaluation of the prognostic significance of vascular endothelial growth factor in node positive primary breast carcinoma. Int J Oncol.

[16] Bruni D, Angell HK, Galon J (2020). The immune contexture and immunoscore in cancer prognosis and therapeutic efficacy. Nat Rev Cancer.

[17] Lee T, Teng TZJ, Shelat VG (2020). Carbohydrate antigen 19 – 9 - tumor marker: past, present, and future. World J Gastrointest Surg.

[18] Mola S, Pandolfo C, Sica A, Porta C (2020). The macrophages-Microbiota Interplay in Colorectal Cancer (CRC)-Related inflammation: prognostic and therapeutic significance. Int J Mol Sci.

[19] Jagieła J, Bartnicki P, Rysz J (2021). Nephrotoxicity as a complication of Chemotherapy and Immunotherapy in the treatment of Colorectal Cancer, Melanoma and Non-small Cell Lung Cancer. Int J Mol Sci.

[20] Kasprzak A (2020). Angiogenesis-related functions of wnt signaling in colorectal carcinogenesis. Cancers (Basel).

